# Rapid Synthesis and Sintering of Metals from Powders

**DOI:** 10.1002/advs.202004229

**Published:** 2021-03-08

**Authors:** Chengwei Wang, Wei Zhong, Weiwei Ping, Zhiwei Lin, Ruiliu Wang, Jiaqi Dai, Miao Guo, Wei Xiong, Ji‐Cheng Zhao, Liangbing Hu

**Affiliations:** ^1^ Department of Materials Science and Engineering University of Maryland College Park College Park MD 20742 USA; ^2^ Department of Mechanical Engineering and Materials Science University of Pittsburgh Pittsburgh PA 15261 USA; ^3^ Center for Materials Innovation University of Maryland College Park College Park MD 20742 USA

**Keywords:** intermetallic compounds, powder metallurgy, refractory metals, ultrafast sintering

## Abstract

Powder to bulk processes, such as additive manufacturing and metal injection molding (MIM), have enabled great potential for complex metal designing and manufacturing. However, additive manufacturing process normally introduces a high residue stress and textures due to the locally intense temperature. MIM is an excellent batch manufacturing process; nevertheless, it is not suitable for rapid screening and development of new metal compositions and structures due to the slow sintering process. Herein, an ultrafast high‐temperature sintering (UHS) process is reported that enables the rapid synthesis and sintering of bulk metals/alloys and intermetallic compounds. In this process, elemental powders are mixed and pressed into pellets, followed by UHS sintering in just seconds at a temperature between 1000 and 3000 °C. Three representative compositions, including pure metals, intermetallics, and multielement alloys, are demonstrated with a broad range of melting points. The UHS process for metal sintering is nonmaterials specific, in addition to being extremely rapid, which make it suitable for materials discovery. Furthermore, the sintering method does not apply pressure to the samples, making it compatible with 3D printing and other additive manufacturing processes of complex structures. This rapid sintering technique will greatly facilitate the development and manufacturing of metals and alloys.

## Graphical abstract

An ultrafast high‐temperature sintering (UHS) process is developed to sinter a wide range of metals/alloys from compacts of elemental or prealloyed powders. Joule‐heated carbon heaters provide a uniform high‐temperature environment (up to 3000 °C) for a rapid sintering in just ≈10 s.

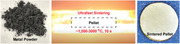

Powder metallurgy is a widely used technology to manufacture metal components from their powders. Unlike traditional metal processing techniques (e.g., casting), powder metallurgy does not require the metal to fully melt, making it especially suitable for the manufacturing of high‐temperature materials.^[^ ^1^, ^2^, ^3^ ^]^ Traditional powder metallurgy, such as metal injection molding (MIM), generally uses a bulk furnace to sinter a pressed green body into a dense component, which requires a long sintering time of hours.^[^ ^4^, ^5^, ^6^ ^]^ Recently, electric current assisted sintering techniques,^[^ ^7^ ^]^ including SPS,^[^ ^8^ ^]^ flash sintering,^[^ ^9^, ^10^, ^11^ ^]^ hot pressing,^[^ ^12^ ^]^ and electro sinter forging,^[^ ^13^, ^14^ ^]^ have been applied to rapidly sinter metals and ceramics with significantly reduced sintering time. However, these methods normally require special dies to hold the samples, which limits the sample size and geometry as well as the application for complex 3D structures. To ensure a good electrical contact between the graphite die and sample pellets, a slight of conductive ink or metal electrodes (e.g., Pt) is normally required,^[^ ^9^, ^15^ ^]^ which further complicated the sintering process. Additionally, when sintering metallic samples, the high current through the sample can cause the current‐induced nonuniform structures.^[^ ^15^ ^]^

Directed energy deposition (DED), a metal additive manufacturing process based on laser/arc metal deposition,^[^ ^16^ ^]^ has been widely used for manufacturing metal or alloy components with complex structures.^[^ ^17^ ^]^ Nevertheless, laser melting and electric current assisted sintering also generate physical textures on the materials due to the locally intense temperature,^[^ ^18^, ^19^, ^20^ ^]^ leading to unwanted anisotropic properties that are difficult to remove.^[^ ^21^ ^]^ Furthermore, DED and other powder‐based additive manufacturing processes usually require high‐quality spherical powders with a specific size distribution, which increases the cost and severely limits their wide application. Clearly, there is a need for a universal, rapid sintering technique for powder metallurgy.

Recently, we developed an ultrafast high‐temperature sintering (UHS) method that can quickly synthesize and sinter ceramics (e.g., solid state oxide electrolytes) directly from precursor powders in just ≈10 s.^[^ ^22^ ^]^ In this study, we extend the UHS method to rapidly sinter a wide range of metals/alloys from compacts of elemental or prealloyed powders (**Figure** **1**a). In a typical UHS process, a green body is closely sandwiched by two carbon heaters, which through Joule heating provide a uniform high‐temperature environment (up to 3000 °C) for a uniform sintering. The resulting ultrahigh temperature can rapidly sinter a broad range of metals, including those with particularly high melting points, resulting in pellets with thicknesses of ≈1 mm that feature a shiny and dense structure after brief polishing (Figure 1b). The highly controllable short processing time (10–30 s) enables excellent control of the composition and microstructure of the sintered material. As a proof‐of‐concept, we successfully sintered a wide range of metals and alloys, including Cu, steel, refractory metals, and intermetallics. In our UHS technique, no special dies are needed to hold the sample, and the current through the sample pellet is negligible. Moreover, the thin (about 2 mm) heating section of the carbon felt enables fast heating and cooling, which benefits fine grains and therefore better mechanical performances. Additionally, our UHS technique also enables real‐time, direct observation of the sintering process, which allows in situ and rapid adjustment of sintering temperature and time to achieve a good sintering. It is also a key feature allowing us to better study the sintering mechanisms. This technique can provide a universal rapid sintering for powder metallurgy and greatly accelerate the development of alloy materials and structures.

**Figure 1:**
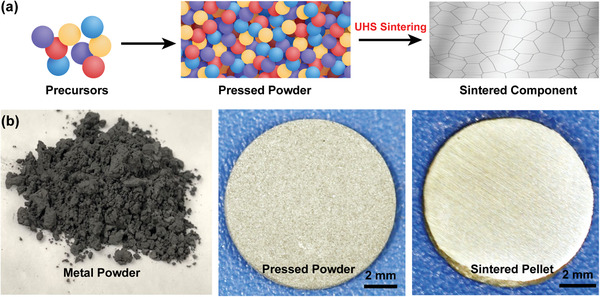
a) Schematic and b) corresponding photographs of the UHS process for rapid sintering of metals and alloys directly from pressed precursor powders.

A typical setup of our UHS technique is shown in **Figure** **2**a–c, where two carbon strips are used as the Joule‐heaters to sandwich the samples. The temperature of the carbon heater (Figure 2a) can rapidly increase up to 1500–3000 °C (Figure 2b) in ≈10 s. The pressed precursor pellet is in close contact with the carbon heaters (Figure 2c), which can achieve heating rates of up to 10^3^–10^4^ °C min^−1^ (Figure 2d), ≈2–3 orders of magnitude higher than that of conventional furnace sintering. The high sintering temperature (up to 3000 °C) greatly accelerates the diffusion and reaction processes of the precursor elements, which enables rapid sintering in just seconds. In addition, the ultrafast cooling rate of 10^3^–10^4^ °C min^−1^ can help avoid phase separation and abnormal grain growth during cooling to achieve the targeted alloy phases with the designed structure. For a typical carbon heater with a size of about 10 cm × 1 cm × 3 mm, the temperature‐power profile is shown in Figure 2e. Note that the center of the heater was cut out to form a space of about 1 cm × 1 cm × 1 mm for sample, and the temperature was measured from this area. UHS can provide well‐controlled sintering temperatures from ≈1000 to ≈3000 °C (Figure 2f) to rapidly sinter a wide range of metals and alloys, including Cu, steel, W, and intermetallics, directly from mixtures of the corresponding elemental powders (Figure 2g). The melting temperatures of these metals and alloys vary from ≈1000 to ≈3400 °C and yet both single and multielemental compositions can be sintered. The resulting sintered materials, including Cu, stainless steel, pure W (with a melting point of 3422 °C), and Nb_5_Si_3_, and MoSi_2_ silicides, exhibit a rigid structure with a shiny surface after polishing, demonstrating the universality of the UHS technique for rapid metal sintering (Figure 2g).

**Figure 2:**
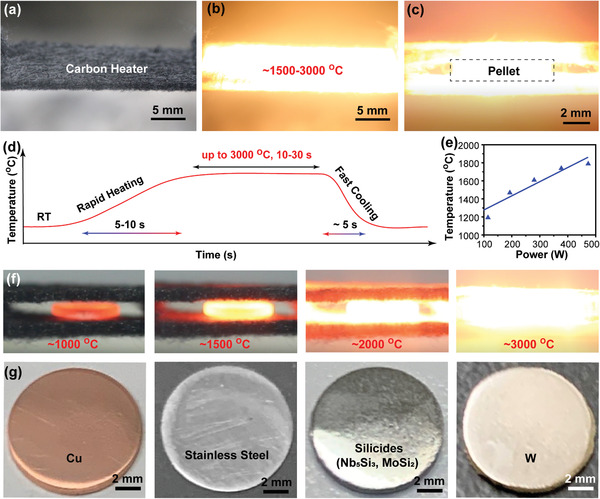
Typical photographs of the heater at a) room temperature and b) ≈1500–3000 °C. c) The sample pellet sandwiched by two carbon heaters during UHS sintering. d) Schematic temperature profile of a UHS run. e) The typical temperature‐power profile of the carbon heater with a size of about 10 cm × 1 cm × 3 mm. f) The UHS sintering process at temperatures of ≈1000, ≈1500, ≈2000, and ≈3000 °C. g) Photographs of UHS‐sintered metals and alloys, including Cu, stainless steel, silicides such as MoSi_2_ and Nb_5_Si_3_, and W.

The ultrahigh temperature of the carbon heaters can melt or partially melt precursor powders with various melting temperatures in a short time, allowing alloys with complex compositions to be manufactured. We demonstrated this capability by sintering a 30Al‐60Cr‐10Si (at.%) alloy from elemental powders of Al, Cr, and Si, which feature a large difference in their melting points (Cr, 1907 °C; Si, 1414 °C; Al, 660 °C). We can fine tune the sintering temperature in the zone near the melting temperature of Cr (**Figure** **3**b) so that the alloy can partially melt to rapidly react and form different phases. A single UHS run at ≈1700 ± 100 °C for ≈10 s is enough to form the expected Cr_3_Si and two Al_8_Cr_5_ phases (Figure 3c). Scanning electron microscopy (SEM) indicates the sintered pellet features a dense structure with the different alloy phases (Figure 3d). The energy‐dispersive X‐ray spectroscopy (EDS) mapping results (Figure 3e–g) indicate the precursor elements have thoroughly diffused and reacted to form the alloy phases during the rapid UHS process. Note that no secondary oxides were observed due to the inert atmosphere and the use of the carbon felt as a heating element. The short sintering time also prevented the carburization of the carbon felt. Therefore, the UHS process enables fast diffusion, liquid phase/reactive sintering at high temperature to achieve alloying and consolidation in a single step. This type of combined process can be used to synthesize and sinter pure metal, alloy, or intermetallic compound.

**Figure 3:**
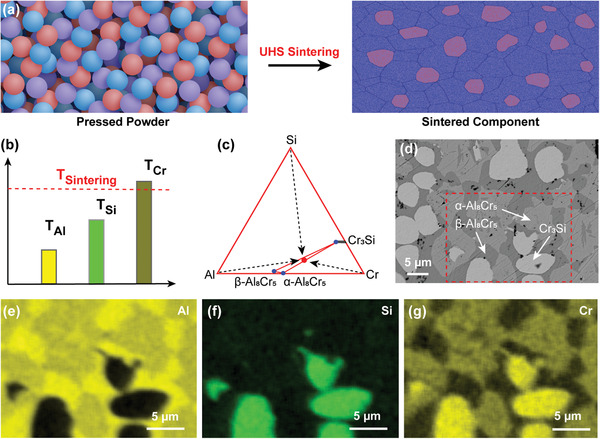
a) Schematic of the UHS process for a three‐element 30Al‐60Cr‐10Si (at.%) alloy. b) Schematic of the UHS sintering temperature relative to the melting points of the elements. c) Schematic showing the alloy and phase formation from elemental powders. d) SEM backscattered electron image for a 30Al‐60Cr‐10Si (at.%) alloy made from the corresponding elemental powders via UHS. e–g) EDS elemental maps showing the formation of the Cr_3_Si phase and two Al_8_Cr_5_ phases with slightly different Al and Cr contents.

We further extended the UHS method to sinter intermetallic compounds, which possess excellent mechanical properties at high temperature, but are difficult to manufacture due to the high melting points.^[^ ^23^ ^]^ **Figure** **4** shows the synthesis of MoSi_2_ (melting point of 2020 °C) and Nb_5_Si_3_ (melting point of 2515 °C) from the pressed elemental powders via UHS in ≈10 s at ≈1800 ± 100 and ≈2300 ± 100 °C, respectively. For the MoSi_2_ case, local composition variations in the powder pellet led to the formation of a small amount of Mo_5_Si_3_ and un‐reacted pure Si (Figure 4b,c). A slightly higher temperature or longer sintering time will help their reaction to form monolithic MoSi_2_. For the Nb_5_Si_3_ case, there were some residual eutectic (Figure 4e,f), which can also be further reacted to form the monolithic Nb_5_Si_3_ phase.

**Figure 4:**
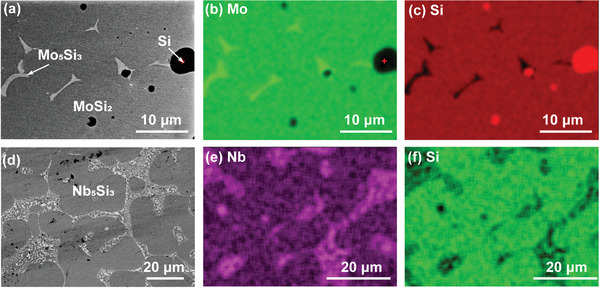
SEM backscattered electron images and EDS element maps of a–c) MoSi_2_ and d–f) Nb_5_Si_3_ made from elemental powders by UHS, showing the formation of the MoSi_2_ phase (the predominant phase) together with a small amount of the Mo_5_Si_3_ phase and some residual pure Si as well as the Nb_5_Si_3_ phase (the predominant phase) and some residual eutectic microstructure. The EDS maps (e) and (f) were taken from different areas of the sample and at a different magnification from the SEM image area of (d).

It is worth noting that the pressed pellet of Mo and Si powders went through a self‐propagating exothermic reaction at ≈1200 °C to form MoSi_2_ during the UHS heat up process, which led to an uncontrollable sintering and a relatively porous sample.^[^ ^24^, ^25^ ^]^ The sample was then ground to powder, compressed into a green pellet and re‐sintered to form a dense MoSi_2_ sample. The Nb_5_Si_3_ sample was prepared in the same fashion. We also note that monolithic bulk Nb_5_Si_3_ is difficult to achieve a high density by conventional sintering due to the high melting point (2515 °C). Meanwhile, arc‐melting of Nb_5_Si_3_ from Nb and Si chunks usually requires more than three times to achieve homogeneity.^[^ ^26^ ^]^ The arc‐melted sample would be much like the microstructure in Figure 4d with residual eutectic regions that can be homogenized away by a post high‐temperature heat treatment.^[^ ^26^ ^]^

The rapid synthesis and sintering enabled by fast element diffusion at high temperature allows us to sinter alloys with more complex compositions without the constraint of the eutectic or near eutectic compositions that are confined by the casting route. As a demonstration, we successfully synthesized and sintered a complex 7‐element Nb‐based alloy of 41.9Nb‐22.5Ti‐20.9Si‐9.2Cr‐2Al‐2B‐1.5Sn (at.%) (**Figure** **5**). The melting point of the elemental powders varied significantly, from 450 °C (Sn) to 2477 °C (Nb), yet UHS at ≈2200 ± 100 °C for 30 s was able to form the alloy consisting of the expected phases: bcc, Nb_5_Si_3_, Nb(Cr,Si)_2_, and TiB_2_ (melting point, 3230 °C). In the SEM image of the resulting compound (Figure 5a), the bright phase is the Nb‐based bcc solid‐solution with Al and Sn; the predominant gray phase is Nb_5_Si_3_ with Ti and a small amount of Cr and Al; the dark gray phase is the C‐14 Laves phase Nb(Cr,Si)_2_ with Ti and Al; and the dark phase is TiB_2_. During the UHS heating up process, this alloy also experienced a milder self‐propagating exothermic reaction at ≈1200 °C, leading to a relatively porous sample, which we then ground into powder and re‐sintered to achieve a dense alloy. We were also able to make a relatively dense sample of this alloy using a single UHS run by carefully controlling the UHS heating process to avoid the self‐propagating exothermic reaction.

**Figure 5:**
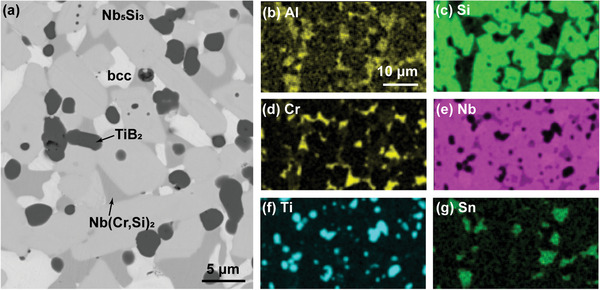
a) SEM backscattered electron image and b–g) EDS element maps for a complex Nb‐based alloy (41.9Nb‐22.5Ti‐20.9Si‐9.2Cr‐2Al‐2B‐1.5Sn, at.%) made from elemental powders by UHS. The results show the formation of four different phases with the Sn‐containing and Nb‐rich phase being bcc, the Si‐rich phase being Nb_5_Si_3_ (the predominant phase), the Cr‐rich phase being the C‐14 Laves phase Nb(Cr,Si)_2_, and the Ti‐rich phase being TiB_2_. The EDS maps were taken from a different area of the sample than the SEM image in (a) and at a different magnification.

We note that the predominant Nb_5_Si_3_ phase is quite fine—on the order of a dozen microns—which is an order of magnitude smaller than that of similar alloys in the cast version.^[^ ^27^, ^28^ ^]^ We would expect the finer microstructure of the silicide phase to increase both the mechanical properties and oxidation resistance of the material. UHS also allows a higher volume fraction of the Nb_5_Si_3_ phase to be designed to Nb silicide composites without the constraint of the eutectic or near eutectic compositions confined by the casting route. It is also noted that the Nb silicide composite alloy has a eutectic point around 1700 °C, which is difficult to cast without specially engineered cast molds.^[^ ^27^, ^28^ ^]^ The UHS process renders such high‐melting temperature alloys much easier to make without the need for high‐temperature furnaces or molds.

For the first time, we have successfully applied the UHS technique to rapidly sinter metals and alloys directly from pressed powders in just seconds. Because of the highly tunable sintering temperature of the UHS technique, metals, and alloys with a wide range of melting temperatures can be synthesized and sintered. The short sintering time also allows the fabrication of multicomponent alloys from precursor elements without phase separation. The highly controllable sintering temperature and time also enable excellent control of the material composition and microstructure. As a demonstration, we have sintered pure metals, intermetallics, and alloys with complex compositional designs. Furthermore, we employed a wide range of powder sizes (≈1–50 µm) in the UHS synthesis of the demonstrated metals, alloys, and intermetallics, making this technique far more versatile than DED. For the specially designed silicide composite material, the small, uniformly distributed silicide grains enabled by UHS rapid sintering demonstrate great potential for high‐temperature applications. We expect this universal, rapid sintering technique to accelerate the development of high performance metals and alloys, particularly for high‐temperature materials that are difficult to manufacture by casting.

## Experimental Section

##### UHS Setup

The UHS setup is similar as that described in the previous work.^[^ ^22^ ^]^ Specifically, the carbon heaters were made from carbon felt (AvCarb Felt G200, Fuel Cell Store) with a size of ≈10 cm x 1 cm x 3 mm. The center of the heater was cut out to form a space of about 1 cm × 1 cm × 1 mm for sample. By employing two steel clips, the carbon heaters were connected to a high‐power DC source (Volteq HY6020EX) with tunable current (0–20 A) and voltage (0–50 V). The sintering process was performed in argon atmosphere.

##### Synthesis and Sintering of Metals/Alloys from Elemental Powders

The powders were purchased from Atlantic Equipment Engineers, including: Al (99.9 wt.%, 1–5 µm), B (99.0 wt.%, 1 µm), Cr (99.5 wt.%, 7–9 µm), Cu (99.9 wt.%, 1–5 µm), Mo (99.98 wt.%, 1–2 µm), Nb (99.8 wt.%, 1–5 µm), Si (99+ wt.%, 1–5 µm), Sn (99.9 wt.%, 1–5 µm), Ti (99.7 wt.%, < 20 µm), and W (99.9 wt.%, 325 mesh). Stoichiometric amounts of the elemental powders were uniformly mixed by milling for 30 min. Then the mixed alloy precursor powder was uniaxially pressed into disks (6–10 mm in diameter, ≈0.5 mm in thickness) under a pressure of 3 MPa. To improve the sintering quality, some pressed disks were further pressed with a cold isostatic pressing machine under a pressure of ≈40 MPa. The pressed green pellets were sintered by UHS for 10–30 s to fabricate the alloys with a relative density of > 95%. For the silicides, the elemental precursors normally went through fast self‐propagating exothermic reactions at ≈1200 °C, which can result in the melting of the pellet and a relatively porous structure. To obtain a dense pellet with maintained shape, the reacted pellets were regrained to powder and then pressed into pellets for a second round of UHS sintering. Alternatively, the pressed green pellets can react at ≈800–1000 °C for about 1 min to slowly finish the reactions, then go through UHS sintering at high temperature in one step.

##### Characterization

Morphology and elemental mapping analysis of the samples were performed by a Hitachi SU‐70 FEG‐SEM with an EDS detector under an accelerating voltage of 10–15 kV. Before conducting SEM, the sintered alloy pellets were cold mounted and ground by silicon carbide sandpaper and then polished to a 1 µm surface furnishing using diamond paste. The phase structure of the alloy was determined by X‐ray diffraction using a Cu K*α* radiation source (*λ* = 1.54056 Å) at 40 kV and 40 mA (Bruker D8 Advance powder diffractometer). The sintering temperatures of the heater were measured following the previous method by a specially designed pyrometer.^[^ ^29^ ^]^ Specifically, a Vision Research Phantom Miro M110 high‐speed camera was used to capture the emission spectra, which were fitted using the Planck function to obtain temperatures. The pyrometer was placed outside the glove box.

## Conflict of Interest

The authors declare no conflict of interest.
